# Enterogenous cyst of pediatric testis: a case report

**DOI:** 10.1186/s13256-017-1372-6

**Published:** 2017-07-30

**Authors:** Yoko Saitoh, Takashi Kawahara, Masako Otani, Yohei Kumano, Masahiro Yao, Jun-ichi Teranishi, Hiroji Uemura

**Affiliations:** 1 Departments of Urology and Renal Transplantation, Yokohama City University Medical Center, Yokohama, Japan; 2 Department of Urology, Yokohama City University Graduate School of Medicine, Yokohama, Japan; 3 Division of Diagnostic Pathology, Yokohama City University Medical Center, Yokohama, Japan

**Keywords:** Pediatric testicular tumor, Enterogenous cyst

## Abstract

**Background:**

An enterogenous cyst is a rare entity categorized as an intestinal cyst. In most cases, enterogenous cysts are seen in the mediastinum, peritoneal cavity, spinal canal, subarachnoid space, and cerebral ventricle.

**Case presentation:**

A 14-year-old Asian (Japanese) boy reported feeling pain in his left groin, and a mass was found. We did not perform orchiectomy because intraoperative frozen sections showed no malignant findings. On histological examination the resected specimens contained columnar epithelium surrounded by smooth muscle. Based on these findings, an enterogenous cyst was diagnosed. Few cases of enterogenous cysts of the testis have been described.

**Conclusion:**

We encountered a case of an enterogenous cyst of a pediatric testis.

## Background

An enterogenous cyst is a rare entity categorized as an intestinal cyst. On histological examination, enterogenous cysts have intestinal epithelium and smooth muscle [1–3]. In most cases, enterogenous cysts are seen in the mediastinum, peritoneal cavity, spinal canal, subarachnoid space, and cerebral ventricle [1–3]. There are few reported cases of enterogenous cyst of the testis [3]. Here we report a case of an enterogenous cyst of the pediatric testis which was successfully treated with preservation of the testis.

## Case presentation

A 14-year-old Asian (Japanese) boy was referred to our department for further examination of a left testicular tumor, which had been detected after he presented with sudden pain in his groin 5 days earlier. His left testis showed no swelling and no rubefaction. Four days after the initial visit, he underwent a surgical operation on his left testicular tumor. He had no remarkable medical history including past medical, social, family, and environmental history, except for mild mental retardation.

Ultrasonography (US) showed irregular contrast in his testicular tumor. The laboratory data were nearly within normal limits, including alpha-fetoprotein (AFP) and human chorionic gonadotropin (hCG): 1.0 ng/mL and <0.1 mIU/mL, respectively. His lactate dehydrogenase (LDH) was slightly elevated at 226 U/L. His laboratory data for complete blood cell counts and serum examination were as follows: white blood cell (WBC) 5590/μL, red blood cell (RBC) 483/μL, platelets (Plt) 28.8/μL, aspartate aminotransferase (AST) 17 IU/L, alanine aminotransferase (ALT) 9 IU/L, and creatinine 0.43 mg/dL. A urine analysis showed no remarkable findings. Physical and neurological examinations showed normal findings, and his blood pressure was 110/62 mmHg at the time of admission. Because of these findings, we could not rule out teratoma or other testicular malignancies, so a rapid pathological examination during surgery was performed. He was found to be free from recurrence 12 months after surgery using US.

### Operative course

We first performed US to detect the location of the tumor and cut the testicular tunica, after which an additional 4-cm incision was made on his central scrotum. Although the tumor margin was not detected, we extracted the testicular tumor with the surrounding normal testis. The tumor had multiple capsuled cysts containing liquid. An intraoperative rapid pathological diagnosis showed no malignant findings, so we did not perform orchiectomy. On histological examination, the tumor showed spermatogenesis and consisted of intestinal tissue.

### Pathological findings

**Fig. 1:**
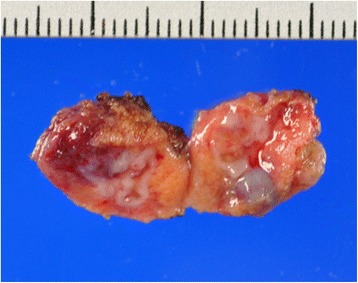
Resected testis specimens

**Fig. 2:**
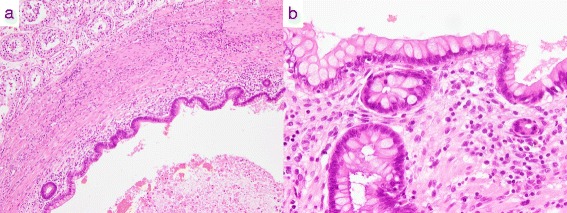
Hematoxylin and eosin staining in **a** low-power and **b** high-power field

The resected specimen was 12×11×10 mm in size, including the tumor and marginal tissue (Fig. 1). On histological examination, the tumor consisted of cystic glands covered by columnar epithelium, occasionally mixed with goblet cells and surrounded by a smooth muscle layer. Partial calcification was observed. The specimen contained no other elements of germ layers except for columnar epithelium without atypia and smooth muscle. Based on these findings, the tumor was diagnosed as an enterogenous cyst (Fig. 2a, b).

## Discussion

An enterogenous cyst is an intestinal cyst with muscle layer cells and cysts covered by intestinal cysts. Several hypotheses regarding the development of this tumor have been proposed. One hypothesis suggests that the spine and endoderm were differentiated insufficiently during the embryonal period, because enterogenous cysts are usually seen in the central nervous system or as spinal abnormalities [1]. Another hypothesis suggests that partial lesions due to ischemia develop into enterogenous cysts, because this tumor is sometimes seen with closure or stricture of the intestine and short-gut syndrome. However, the precise cause of enterogenous cysts is still unknown.

On histological examination, enterogenous cysts contain intestinal epithelium surrounded by smooth muscle, mimicking intestinal mucosa [1–3]. In most cases, these cysts are found in the mediastinum, peritoneal cavity, spinal canal, subarachnoid space, and cerebral ventricle. There have been very few cases of testicular enterogenous cysts. The only other reported case involving an enterogenous cyst of the testis was reported by Mondaini *et al*. in 2006 [1]. Since then, no other cases have been reported. In that case, the patient was a 55-year-old man with left testicular swelling and pain. An enterogenous cyst was diagnosed based on the tumor containing a columnar epithelial lining, smooth muscle layer, and fibrous stroma.

The differential diagnoses of enterogenous cyst include teratoma, metastasis from a mucinous tumor, and mucinous cystadenoma. Most pediatric testicular tumors are mature teratomas, which contain three elements of germ layers such as epithelia, cartilage, muscle, nerve, as well as other types of tissue. A teratoma is defined as a benign tumor and is usually nucleated; the testis can be preserved in some cases, depending on the intraoperative pathological diagnosis [4].

Metastatic mucinous cyst of the testis is more frequently observed than mucinous tumors with a testicular origin [5]. The colon, stomach, and pancreas were found to account for 53% of all metastatic mucinous cysts of the testis. Ulbright and Young reported a similar case of mucinous cystadenoma of the testis with intestinal type disease [6]. However, mucinous cystadenoma shows atypia and does not contain a smooth muscle layer.

This pediatric enterogenous cyst arose solely from the testis with cylindrical epithelium and a smooth muscle layer. Because we observed no findings suggestive of malignancy, the tumor was successfully removed and the testes preserved. Mondaini *et al*. also reported a case of enterogenous cyst removal with successful preservation of the testes and no recurrence for 1 year [1]. Our case also showed no recurrence 1 year after surgery, but careful observation should be performed. Enterogenous cysts are typically seen in the mediastinum and peritoneal cavity, and only one case report of enterogenous cyst of the testis has been published.

## Conclusion

Here we described a case of an enterogenous cyst of the testis in a pediatric patient with successful preservation of the testis.
